# The protective effect of tumor necrosis factor-alpha inhibitors in COVID-19 in patients with inflammatory rheumatic diseases compared to the general population—A comparison of two German registries

**DOI:** 10.3389/fmed.2024.1332716

**Published:** 2024-03-06

**Authors:** Rebecca Hasseli, Frank Hanses, Melanie Stecher, Christof Specker, Tobias Weise, Stefan Borgmann, Martina Hasselberger, Bernd Hertenstein, Martin Hower, Bimba F. Hoyer, Carolin Koll, Andreas Krause, Marie von Lilienfeld-Toal, Hanns-Martin Lorenz, Uta Merle, Susana M. Nunes de Miranda, Mathias W. Pletz, Anne C. Regierer, Jutta G. Richter, Siegbert Rieg, Christoph Roemmele, Maria M. Ruethrich, Tim Schmeiser, Hendrik Schulze-Koops, Anja Strangfeld, Maria J.G.T. Vehreschild, Florian Voit, Reinhard E. Voll, Jörg Janne Vehreschild, Ulf Müller-Ladner, Alexander Pfeil

**Affiliations:** ^1^Section of Rheumatology and Clinical Immunology, Department of Internal Medicine D, University Hospital Münster, Münster, Germany; ^2^Department of Rheumatology and Clinical Immunology, Justus-Liebig University Giessen, Giessen, Germany; ^3^Emergency Department and Department for Infectious Diseases and Infection Control, University Hospital Regensburg, Regensburg, Germany; ^4^Department I of Internal Medicine, University of Cologne, Faculty of Medicine and University Hospital Cologne, Cologne, Germany; ^5^Department of Rheumatology and Clinical Immunology, KEM Kliniken Essen-Mitte, Essen, Germany; ^6^Biocontrol Jena, Jena, Germany; ^7^Department of Infectious Diseases and Infection Control, Ingolstadt Hospital, Ingolstadt, Germany; ^8^Klinikum Passau, Passau, Germany; ^9^Klinikum Bremen-Mitte, Bremen, Germany; ^10^Department of Pneumology, Infectious Diseases, Internal Medicine and Intensive Care, Klinikum Dortmund GmbH, Dortmund, Germany; ^11^Department for Rheumatology and Clinical Immunology, University of Schleswig-Holstein, Kiel, Germany; ^12^Department of Rheumatology, Clinical Immunology and Osteology, Immanuel Hospital Berlin, Berlin, Germany; ^13^Department of Hematology and Medical Oncology, University Hospital Jena, Jena, Germany; ^14^Department of Internal Medicine V, University of Heidelberg, Heidelberg, Germany; ^15^Department of Gastroenterology and Infectious Diseases, Heidelberg University Hospital, Heidelberg, Germany; ^16^Department of Medicine II, University of Freiburg, Freiburg, Germany; ^17^Institute for Infectious Diseases and Infection Control, Jena University Hospital, Jena, Germany; ^18^Epidemiology Unit, German Rheumatism Research Center Berlin, Berlin, Germany; ^19^Department of Rheumatology, University Hospital Düsseldorf, Medical Faculty of Heinrich-Heine-University, Düsseldorf, Germany; ^20^Hiller Research Center, University Hospital Düsseldorf, Medical Faculty of Heinrich-Heine-University, Düsseldorf, Germany; ^21^Division of Infectious Diseases, Department of Medicine II, University of Freiburg, Freiburg, Germany; ^22^Department of Gastroenterology, Faculty of Medicine, University of Augsburg, Augsburg, Germany; ^23^Private Practice, Cologne, Germany; ^24^Division of Rheumatology and Clinical Immunology, Department of Internal Medicine IV, University of Munich, Munich, Germany; ^25^Department of Internal Medicine, Infectious Diseases, University Hospital Frankfurt, Goethe University Frankfurt, Frankfurt am Main, Germany; ^26^Department of Internal Medicine II, School of Medicine, University Hospital Rechts Der Isar, Technical University of Munich, Munich, Germany; ^27^Department of Rheumatology and Clinical Immunology, Faculty of Medicine, Medical Center-University of Freiburg, University of Freiburg, Freiburg, Germany; ^28^Department II of Internal Medicine, Hematology/Oncology, Goethe University, Frankfurt, Germany; ^29^Department of Internal Medicine III, University Hospital Jena, Jena, Germany

**Keywords:** inflammatory rheumatic diseases, COVID-19, general population, tumor necrosis factor-alpha inhibitors, severe disease

## Abstract

**Objectives:**

To investigate, whether inflammatory rheumatic diseases (IRD) inpatients are at higher risk to develop a severe course of SARS-CoV-2 infections compared to the general population, data from the German COVID-19 registry for IRD patients and data from the Lean European Survey on SARS-CoV-2 (LEOSS) infected patients covering inpatients from the general population with SARS-CoV-2 infections were compared.

**Methods:**

4310 (LEOSS registry) and 1139 cases (IRD registry) were collected in general. Data were matched for age and gender. From both registries, 732 matched inpatients (LEOSS registry: *n* = 366 and IRD registry: *n* = 366) were included for analyses in total.

**Results:**

Regarding the COVID-19 associated lethality, no significant difference between both registries was observed. Age > 65°years, chronic obstructive pulmonary disease, diabetes mellitus, rheumatoid arthritis, spondyloarthritis and the use of rituximab were associated with more severe courses of COVID-19. Female gender and the use of tumor necrosis factor-alpha inhibitors (TNF-I) were associated with a better outcome of COVID-19.

**Conclusion:**

Inflammatory rheumatic diseases (IRD) patients have the same risk factors for severe COVID-19 regarding comorbidities compared to the general population without any immune-mediated disease or immunomodulation. The use of rituximab was associated with an increased risk for severe COVID-19. On the other hand, the use of TNF-I was associated with less severe COVID-19 compared to the general population, which might indicate a protective effect of TNF-I against severe COVID-19 disease.

## Highlights:

• The risk of infection with SARS-CoV-2 might be higher in patients with inflammatory rheumatic disease compared to the general population.
• The data of two German nationwide registries revealed no increased COVID-19 associated lethality in inflammatory rheumatic diseases.
• The use of tumor necrosis factor-alpha inhibitors was associated with a better outcome of severe COVID-19 disease (odds ratio of 0.5; 95% CI 0.2 to 0.9).

## Introduction

In December 2019, the first cases of patients with unexplained pneumonia were reported in Wuhan, China (1), which was identified in January 2020 as an infection with SARS-CoV-2. At the same time, first cases of COVID-19 were reported in Germany (2). Since March 2020, a global pandemic SARS-CoV-2 has been declared (2). The highest fatality rate was observed in elderly and multi-morbid persons (3). Patients with autoimmune disease were expected to harbour an increased risk for severe COVID-19, especially under the treatment with glucocorticoids (4, 5). In addition, disease activity of inflammatory rheumatic diseases (IRD) was associated with more severe COVID-19 compared to patients with sustained remission (6). Further, IRD patients under treatment with rituximab revealed a poor outcome of COVID-19 (6–9). For some patient groups (e.g., patients with oncological morbidities), a worse outcome of COVID-19 has been described compared to non-pre-diseased patients (10). Some data suggested an increased risk for IRD-patients to develop severe COVID-19, such as COVID-19 related pneumonia, compared to the general population (11, 12). Especially in patients with rheumatoid arthritis and systemic vasculitis, a higher risk for severe COVID-19 could be observed (12).

As disease activity of IRD plays a crucial role regarding COVID-19, severity and different healthcare systems might have an influence on this aspect. The aim of this study was to investigate whether inpatients with IRD are at higher risk to poor outcomes of COVID-19 compared to the general population in Germany. In addition, the question was whether IRD could be considered as a comorbidity increasing the risk for severe COVID-19 and if so, whether specific types of disease-modifying antirheumatic drugs (DMARD) were contributing factors.

## Materials and methods

Data from the German COVID-19 registry for patients with IRD^1^ and data from the Lean European Open Survey on SARS-CoV-2 infected patients (LEOSS registry)^2^ obtained between March 2020 until January 2021 were analysed. Patients with IRD and SARS-CoV-2 infection were included in the ongoing IRD registry by their rheumatologists (13). The LEOSS cohort includes cases of SARS-CoV-2 infections in the general German population (14). Here data were entered by treating physicians, study nurses and other medical staff. In both registries, participating centres included both academic and non-academic clinics throughout Germany.

**FIGURE 1:**
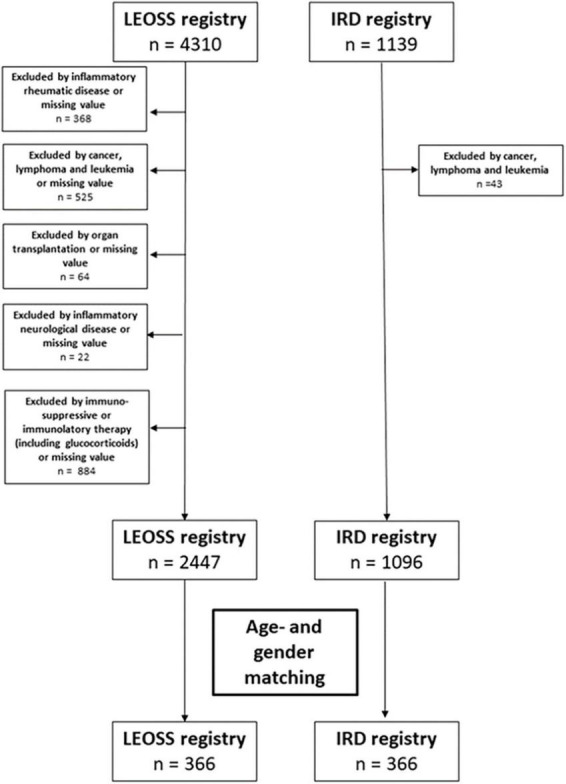
Flowchart regarding the excluded patients or missing value for matching of the patients from both registries.

Data and missing values of patients with immune-mediated inflammatory diseases, cancer, a history of cancer, organ transplantation or immunomodulatory/immunosuppressive treatment (including glucocorticoids) were excluded for further analyses from both cohorts due to the known poorer outcomes of patients with SARS-CoV-2 infection (details see Figure 1). As in LEOSS, mainly COVID-19 inpatients were reported, our analysis focussed on COVID-19 inpatients only from both registries.

For comparison, age-matching and gender-matching were used, as these two variables have been shown to strongly impact on the course of COVID-19. For age and gender a 1:1 matching was performed.

### Statistical analyses

Completed cases were reviewed and queried in case of missing or inconsistent data. Statistical computations were performed using the programming language Python (version 3.10.0) and the additional packages numpy (version 1.22.3), pandas (version 1.4.1), scipy (version 1.8.0), scikit-learn (version 1.1.2), statsmodels (version 0.13.2), matplotlib (version 3.5.1), and seaborn (version 0.11.2). The use of the individual packages is structured as follows: performing basic numerical operations (numpy), processing of tabular data (pandas), testing group differences between both registries (scipy), ordinal logistic regression modeling (statsmodels), multiple imputation of missing values (sklearn) and data visualisation (matplotlib, seaborn).

The respective statistical significance level of group differences between both registries (see Tables 1(5) were calculated using the Mann-Whitney *U* test (scipy.stats.mannwhitneyu) for continuous variables as well as the Chi-Square test (scipy.stats.chi2_contingency) for categorical variables. *P*-values <0.05 were considered significant.

**TABLE 1:** Baseline characterisitca (n. s., not significant; n. a., not applicable).

		German COVID-19 registry (IRD registry)	Lean European open survey on SARS-CoV-2 (LEOSS registry)	Significance
Total		*n* = 366	*n* = 366	*p* = n. s.
Men		*n* = 140	*n* = 140	*p* = n. s.
Age in years				
≤65°years		*n* = 162	*n* = 162	*p* = n. s.
>65 to ≤75°years		*n* = 93	*n* = 93	
>75°years		*n* = 111	*n* = 111	
Height in cm (mean ± standard deviation)		168.6 ± 8.9	n. a. (*n* = 0)	n. a.
Weight in Kg (mean ± standard deviation)		79 ± 17	n. a. (*n* = 0)	n. a.
Body mass index				
<30 kg/m^2^		*n* = 155	*n* = 202	*p* = n. s.
≥30 kg/m^2^		*n* = 81	*n* = 98	
Inflammatory rheumatic diseases	Rheumatoid arthritis	*n* = 196 (53.6%)	−	
	Spondyloarthritis	*n* = 18 (4.9%)	−	
	Psoriatic arthritis	*n* = 23 (6.3%)	−	
	Enteropathic arthritis	*n* = 4 (1.1%)	−	
	Polymyositis	*n* = 8 (2.2%)	−	
	Sjögren’s Syndrome	*n* = 3 (0.8%)	−	
	Systemic lupus erythematosus	*n* = 7 (1.9%)	−	
	Systemic sclerosis	*n* = 11 (3.0%)	−	
	Mixed connective tissue disease	*n* = 2 (0.5%)	−	
	Other connective tissue diseases	*n* = 4 (1.1%)	−	
	Granulomatosis with polyangiitis	*n* = 23 (6.3%)	−	
	Eosinophlic granulomatosis with polyangiitis	*n* = 5 (1.4%)	−	
	Microscopic polyangiitis	*n* = 4 (1.1%)	−	
	Polymyalgia	*n* = 24 (6.6%)	−	
	Giant cell arteriitis	*n* = 17 (4.6%)	−	
	Behçet’s disease	*n* = 5 (1.4%)	−	
	Other vasculitis	*n* = 8 (2.2%)	−	
	Other type of IRD	*n* = 30 (8.2%)	−	
Drugs	Glucocorticoids	*n* = 206 (56.3%)	−	
	csDMARD	*n* = 187 (51.1%)	−	
	TNF-I (adalimumab, certolizumab, infliximab, etanercept, golimumab)	*n* = 37 (10.1%)	−	
	Rituximab	*n* = 39 (10.7%)	−	
	Other cytokine inhibitors (secukinumab, ixekizumab, anakinra, canakinumab, ustekinumab, tocilizumab, sarilumab, abatacept)	*n* = 69 (18.9%)	−	
	JAK-I (baricitinib, tofacitinib, upadacitinib)	*n* = 34 (9.3%)	−	

n. s., not significant; n. a., not applicable.

### Ordinal logistic regression analyses

Within the ordinal logistic regressions (statsmodels.miscmodels.ordinal_model.OrderedModel) the COVID-19 severity status was used as the outcome of interest. The COVID-19 severity status was defined using ascending levels: (0) hospitalisation, (1) oxygen administration/non-invasive ventilation and (2) invasive ventilation / extracorporal membrane oxygenation (ECMO) / death. The escalation in COVID-19 severity is assumed to be equidistant between the levels.

The independent variable “rheumatic disease activity” exhibited a small number of missing values (*n* = 27), which were derived by multiple imputation (sklearn.impute.KNNImputer) (15). The independent variable body mass index “BMI” was not considered in multivariate ordinal logistic regression due to high number of missing values (*n* = 196) in both registries. Multicollinearity between the independent variables was quantified using variance inflation factors (statsmodels.stats.outliers_influence.variance_inflation_factor). All independent variables (*n* = 23) considered in multiple ordinal logistic regression exhibited variance inflation factors <5 (16).

Due to the low prevalence of individual rheumatological diagnoses, selected diagnoses were combined into groups: “Sjögren’s Syndrome,” “Systemic Lupus Erythematosus,” “Systemic Sclerosis,” “Mixed Connective Tissue Disease,” “Polymyositis,” and “Other Connective Tissue diseases” were combined to the “Connective Tissue Diseases (CTD).” “Granulomatosis with Polyangiitis,” “Eosinophilic Granulomatosis with Polyangiitis,” “Microscopic Polyangiitis,” “Giant Cell Arteriitis,” “Polymyalgia Rheumatica,” “Behçet’s Disease,” and “Other Vasculitis” were combined to the “Vasculitides.” “Ankylosing spondylitis,” “Psoriatic arthritis” and “Enteropathic arthritis” were combined to “Spondyloarthritis” (SpA). RA represents the largest group within rheumatologic diagnoses and was included as a separate individual group in the analysis.

**FIGURE 2:**
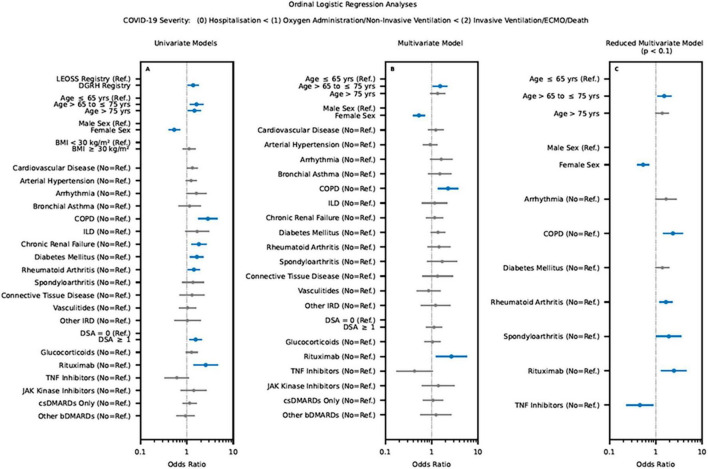
Results of the univariate **(A)** and multivariate regression analysis **(B,C)** for the verification of comorbidities, IRD and rheumatic treatment on the outcome of COVID-19 infection based on the LEOSS Registry and IRD registry. Significant independent variable’s influence (*p*-values <0.05) on the COVID-19 severity outcome marked bold and colored blue. (LEOSS Registry, Lean European Open Survey on SARS-CoV-2; BMI, body mass index; COPD, chronic obstructive pulmonary disease; ILD, interstitial lung disease; IRD, inflammatory rheumatic diseases; DSA, disease activity; bDMARD biological disease-modifying antirheumatic drugs, csDMARD, conventional synthetic disease-modifying antirheumatic drug; TNF Inhibitors, tumor necrosis factor-alpha inhibitors).

Univariate ordinal logistic regression was used to assess the individual association between the COVID-19 severity outcome (dependent variable) and the respective independent variable (Figure 1A). Multivariate ordinal logistic regression was used to assess the independent associations between the COVID-19 severity outcome and all independent variables (*n* = 23) considered in the analysis (Figure 1B). Results were reported as odd ratios (OR) including their respective 95% confidence intervals (CI). *P*-values were reported to describe the significance of the respective independent variable’s influence on the COVID-19 severity outcome. *P*-values <0.05 were considered significant (marked bold and colored blue, see Figure 2).

In order to reduce model complexity, the obtained multivariate ordinal logistic regression model was iteratively reduced by pruning the independent variable exhibiting lowest significance and re-evaluating the reduced model. This backward elimination procedure was repeated until all remaining independent variables exhibited *p*-values <0.1. This threshold was set to retain potentially confounding variables with a strong influence on the model. Only *p*-values <0.05 were considered significant (marked bold and colored blue, see Figure 2).

## Results

### Baseline characteristics

From March 2020 to January 2021, 4310 cases (LEOSS registry) and 1139 cases (IRD registry) of SARS-COV-2 infection were reported. After performing the exclusion criteria and focusing on COVID-19 inpatients, matched pairs by age and gender were developed, consequently 732 patients (LEOSS registry: *n* = 366 and IRD registry: *n* = 366) were subsequently available for analysis.

In the IRD registry, (196/366, 53.6%) of the patients were diagnosed with rheumatoid arthritis, followed by psoriatic arthritis (23/366, 6.3%) and SpA (18/366, 4.9%). The use of glucocorticoids was reported in 206/366 (56.3%) of IRD patients, 51.1% (187/366) were treated with conventional synthetic (cs) DMARDs, 10.1% (37/366) with Tumor Necrosis Factor-alpha Inhibitors (TNF-I), 10.7% (39/366) with rituximab, 18.9% (69/366) with other cytokine inhibitors, and 9.3% (34/366) with Janus kinase inhibitors (JAK-I). At time of SARS-CoV-2 infection, 7.0% (25/366) of IRD patients did not receive any immunomodulatory therapy (see Table 1).

### Comorbidities

Arterial hypertension was the most common comorbidity in both registries (IRD registry: 53.8% vs. LEOSS: 54.4%). Regarding other cardiovascular diseases and arrhythmia, significantly more patients were reported in LEOSS as compared to the IRD registry (cardiovascular diseases: 60.4 vs. 26.8%, arrhythmia: 13.7 vs. 2.5%, *p* < 0.001). The IRD registry revealed significantly more patients with chronic kidney disease (20.8% vs. 13.4%; *p* < 0.05), pulmonary arterial hypertension (3.8% vs. 0.3%; *p* < 0.01), and interstitial lung disease (7.9% vs. 3.6%, *p* < 0.05). Concerning bronchial asthma, chronic obstructive pulmonary disease, liver cirrhosis, the use of alcohol and active smoking, no significant differences between the registries were observed (see Table 2).

**TABLE 2:** Comorbidities.

Comorbidities	German COVID-19 registry (IRD registry) *n* = 366	Lean European open survey on SARS-CoV-2 (LEOSS registry) *n* = 366	Significance of difference
Cardiovascular diseases	*n* = 98 (26.8%)	*n* = 221 (60.4%)	*p* < 0.001
Arterial hypertension	*n* = 197 (53.8%)	*n* = 199 (54.4%)	*p* = n. s.
Arrhythmia	*n* = 9 (2.5%)	*n* = 50 (13.7%)	*p* < 0.001
Bronchial asthma	*n* = 24 (6.6%)	*n* = 19 (5.2%)	*p* = n. s.
Chronic obstructive pulmonary disease	*n* = 38 (10.4%)	*n* = 29 (7.9%)	*p* = n. s.
Interstitial lung diseases	*n* = 29 (7.9%)	*n* = 13 (3.6%)	*p* < 0.05
Chronic kidney disease	*n* = 76 (20.8%)	*n* = 49 (13.4%)	*p* < 0.05
Liver cirrhosis	*n* = 2 (0.5%)	*n* = 0 (0%)	n. a.
Osteoporosis	*n* = 46 (12.6%)	*n* = 0 (0%)	n. a.
Pulmonal hypertension	*n* = 14 (3.8%)	*n* = 1 (0.3%)	*p* < 0.01
Use of alcohol	n = 10 (2.7%)	*n* = 0 (0%)	n. a.
Active smoking	*n* = 26 (7.1%)	*n* = 20 (5.5%)	*p* = n. s.
Use of E-cigarettes	*n* = 0 (0%)	*n* = 13 (3.6%)	n. a.

n. s., not significant; n. a., not applicable.

### COVID-19 related symptoms

In IRD patients, significantly more COVID-19 related symptoms were reported compared to the general population (Table 3). Most commonly were fever (IRD registry 70.2%, LEOSS registry 38.8%; *p* < 0.001), cough (IRD registry 62.3%, LEOSS registry 39.6%; *p* < 0.001), dyspnoea (IRD registry 55.2%, LEOSS registry 30.6%; *p* < 0.001), myalgia (IRD registry 27.9%, LEOSS registry 7.9%; *p* < 0.001), loss of appetite (IRD registry 24.6%, LEOSS registry 6.3%; *p* < 0.001), dysgeusia (IRD registry 19.9%, LEOSS registry 6.6%; *p* < 0.001), headache (IRD registry 18.0%, LEOSS registry 9.3%; *p* < 0.001), diarrhoea (IRD registry 15.8%, LEOSS registry 9.6%; *p* < 0.05), and anosmia (IRD registry 15.6%, LEOSS registry 4.9%; *p* < 0.001) (see Table 3).

**TABLE 3:** Symptoms of COVID-19-infection.

COVID-19 related symptoms	German COVID-19 registry (IRD registry) *n* = 366	Lean European open survey on SARS-CoV-2 (LEOSS registry) *n* = 366	Significance of difference
Fever	*n* = 257 (70.2%)	*n* = 142 (38.8%)	*p* < 0.001
Cough	*n* = 228 (62.3%)	*n* = 145 (39.6%)	*p* < 0.001
Expectoration	*n* = 59 (16.1%)	*n* = 33 (9.0%)	*p* < 0.01
Myalgia	*n* = 102 (27.9%)	*n* = 29 (7.9%)	*p* < 0.001
Headache	*n* = 66 (18.0%)	*n* = 34 (9.3%)	*p* < 0.001
Vertigo	*n* = 46 (12.6%)	*n* = 0 (0%)	n. a.
Dyspnoea	*n* = 202 (55.2%)	*n* = 112 (30.6%)	*p* < 0.001
Abdominal pain	*n* = 28 (7.7%)	*n* = 0 (0%)	n. a.
Diarrhoea	*n* = 58 (15.8%)	*n* = 35 (9.6%)	*p* < 0.05
Vomiting	*n* = 35 (9.6%)	*n* = 30 (8.2%)	*p* = n. s.
Loss of appetite	*n* = 90 (24.6%)	*n* = 23 (6.3%)	*p* < 0.001
Anosmia	*n* = 57 (15.6%)	*n* = 18 (4.9%)	*p* < 0.001
Dysgeusia	*n* = 73 (19.9%)	*n* = 24 (6.6%)	*p* < 0.001
Rhinitis	*n* = 30 (8.2%)	*n* = 0 (0%)	n. a.

n. s., not significant; n. a., not applicable

### COVID-19 related complications

The following complications were documented in the IRD registry: 16.9% concomitant infection, 13.7% acute respiratory distress syndrome and 9.0% sepsis. In the LEOSS registry, primarily concomitant infections (14.8%) were reported (see Table 4).

**TABLE 4:** Complications of COVID-19-infection.

COVID-19 related complications	German COVID-19 registry (IRD registry) *n* = 366	Lean European open survey on SARS-CoV-2 (LEOSS registry) *n* = 366	Significance
Acute respiratory distress syndrome	*n* = 50 (13.7%)	*n* = 0 (0%)	n. a.
Sepsis	*n* = 33 (9.0%)	*n* = 0 (0%)	n. a.
Myocarditis	*n* = 5 (1.4%)	*n* = 0 (0%)	n. a.
Heart failure	*n* = 10 (2.7%)	*n* = 0 (0%)	n. a.
Arrhythmia	*n* = 2 (0.5%)	*n* = 4 (1.1%)	*p* = n. s.
Concomitant infection	*n* = 62 (16.9%)	*n* = 54 (14.8%)	*p* = n. s.
Cytokine storm	*n* = 9 (2.5%)	*n* = 0 (0%)	n. a.
Thromboembolism	*n* = 5 (1.4%)	*n* = 13 (3.6%)	*p* = n. s.

n. s., not significant; n. a., not applicable.

### COVID-19 related lethality

Regarding COVID-19 related lethality, no significant difference could be observed in both registries (IRD registry 16.9%, LEOSS registry 15.0%; *p* = n.s., Table 5). Oxygen administration and non-invasive ventilation (NIV) were reported significantly (*p* < 0.001) more frequent in IRD patients (oxygen administration 72.1% and NIV 11.5%) compared to data of the LEOSS registry (oxygen administration 51.6% and NIV 1.1%). Concerning invasive ventilation (IRD registry 18.3%; LEOSS registry 15.3%; *p* = n.s.) and ECMO (each 3.8%), no significant differences were found (see Table 5).

**TABLE 5:** COVID-19 related severe complications.

COVID-19 related severe complications	German COVID-19 registry (IRD registry)	Lean European open survey on SARS-CoV-2 (LEOSS registry)	Significance
Death	*n* = 62 (16.9%)	*n* = 55 (15.0%)	*p* = n. s.
Oxygen administration	*n* = 264 (72.1%)	*n* = 189 (51.6%)	*p* < 0.001
Non-Invasive ventilation	*n* = 42 (11.5%)	*n* = 4 (1.1%)	*p* < 0.001
Invasive ventilation	*n* = 67 (18.3%)	*n* = 56 (15.3%)	*p* = n. s.
Extracorporeal membrane oxygenation	*n* = 12 (3.3%)	*n* = 14 (3.8%)	*p* = n. s.

n. s., not significant.

### COVID-19 outcome analysis

In the univariate ordinal logistic regression, age > 65°years [odds ratio (OR) of 1.6, 95% confidence interval (CI) 1.1 to 2.2], cardiovascular disease (OR of 1.3; 95% CI 1.0 to 1.7), arrhythmia (OR of 1.6; 95% CI 1.0 to 2.6), chronic obstructive pulmonary disease (OR of 2.8; 95% CI 1.7 to 4.6), chronic renal failure (OR of 1.8; 95% CI 1.2 to 2.6), diabetes mellitus (OR of 1.6; 95% CI 1.2 to 2.3) and rituximab (OR of 2.5; 95% CI 1.4 to 4.7) were significantly associated with severe COVID-19. Female patients (OR of 0.5; 95% CI 0.4 to 0.7) showed a significant lower risk for severe COVID-19 compared to males. Regarding arterial hypertension, bronchial asthma, interstitial lung diseases, active smoking, type of IRD, IRD disease activity, use of glucocorticoids, TNF-I, JAK-I, csDMARDs (monotherapy), and other cytokine inhibitors [biological (b) DMARD], no significant association to COVID-19 severity could be identified (see Figure 2A). Compared to sustained remission/low disease activity, moderate/high disease activity was associated with an increased risk for poor COVID-19 outcome (OR of 1.6; 95% 1.2 to 2.1).

Multivariate ordinal logistic regression (excluding body mass index) revealed a significant increased risk for severe COVID-19 in older patients (age > 65 years: OR of 1.5, 95% CI 1.1 to 2.2), patients with chronic obstructive pulmonary disease (OR of 2.3; 95% CI 1.4 to 3.7) or patients treated with rituximab (OR of 2.6; 95% CI 1.3 to 5.1) (see Figure 2B). On the other hand, female gender was associated with lower risk for severe COVID-19 (OR of 0.5; 95% CI 0.4 to 0.7). Although univariate logistic regression model showed an increased association with severe COVID-19 in the case of moderate/high disease activity, this was not statistically confirmed in the multivariate ordinal logistic regression (OR of 1.1, 95% CI 0.8 to 1.7).

Regarding obtained multivariate ordinal logistic regression model after stepwise elimination, similar results could be detected regarding the impact of age (age > 65 years: OR of 1.5, 95% CI 1.1 to 2.1) and chronic obstructive pulmonary disease (OR of 2.3; 95% CI 1.4 to 3.8). In addition, further factors associated with severe COVID-19 were identified: chronic obstructive pulmonary disease (OR of 2.3; 95% CI 1.4 to 3.8), rheumatoid arthritis (OR of 1.6; 95% CI 1.2 to 2.3), SpA (OR of 1.9; 95% CI 1.0 to 3.5) and the use of rituximab (OR of 2.4; 95% CI 1.3 to 4.6). In contrast, female gender (OR of 0.5; 95% CI 0.4 to 0.7) and the use of TNF-I (OR of 0.5; 95% CI 0.2 to 0.9) were associated with a better outcome of COVID-19 inpatients (see Figure 2C).

### COVID-19 outcome sub-analysis inflammatory rheumatic diseases

**FIGURE 3:**
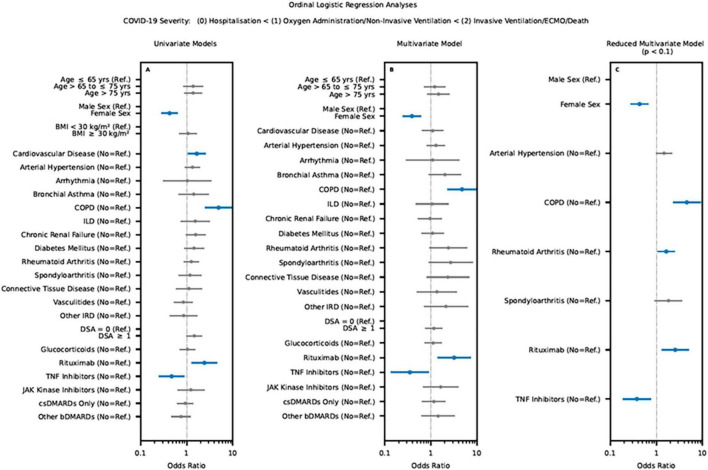
Results of the univariate **(A)** and multivariate regression **(B,C)** sub-analysis for the IRD cohort on the outcome of COVID-19 infection using the data of the IRD registry (significant variable were marked bold and colored blue. LEOSS Registry, Lean European Open Survey on SARS-CoV-2; BMI, body mass index; COPD, Chronic obstructive pulmonary disease; ILD, interstitial lung disease; IRD, inflammatory rheumatic diseases; DSA, disease activity; bDMARD, biological disease-modifying antirheumatic drugs; csDMARD, conventional synthetic disease-modifying antirheumatic drug; TNF inhibitors, tumor necrosis factor-alpha inhibitors).

In the IRD registry, only cardiovascular disease (OR of 1.3; 95% CI 1.0 to 2.6), chronic obstructive pulmonary disease (OR of 4.9; 95% CI 2.5 to 9.7) and rituximab (OR of 2.4; 95% CI 1.3 to 4.6) were significantly associated with severe COVID-19 in the univariate ordinal logistic regression analysis. The use of TNF-I revealed a significant better COVID-19 outcome (OR of 0.5; 95% CI 0.2 to 0.9) (details see Figure 3A).

An equivalent result was observed for the multivariate model (details see Figure 3B).

Regarding the reduced multivariate model chronic obstructive pulmonary disease (OR of 4.5; 95% CI 2.3 to 9.1), rheumatoid arthritis (OR of 1.6; 95% CI 1.1 to 2.5) and rituximab (OR of 2.6; 95% CI 1.3 to 5.0) showed a poor COVID-19 outcome. A better COVID-19 outcome was observed for female gender (OR of 0.4; 95% CI 0.3 to 0.7) and TNF-I (OR of 0.4; 95% CI 0.2 to 0.8) (see Figure 3C).

## Discussion

The aim of this study was to evaluate whether IRD inpatients harbour a higher risk for a poor outcome of SARS-CoV-2 infections compared to the general population and to add evidence whether immunomodulatory treatment has an impact on the outcome of COVID-19.

For both registries, arterial hypertension was shown to be the most common comorbidity in patients with COVID-19 infection (IRD registry: 53.8% and LEOSS registry: 54.4%). A previous published study showed a lower rate of arterial hypertension (33%) in IRD-patients (4). In our study the comorbidity arterial hypertension had an association with hospitalization. Furthermore, 27.7% of non-hospitalized IRD patients had arterial hypertension compared to 43.6% of the hospitalized ones without invasive and 61.5% of the hospitalized ones with invasive ventilation. Additionally, it should be noted that chronic kidney disease or interstitial lung disease were more frequently detected as comorbidities in IRD patients. Both, chronic kidney disease (odds ratio 1.8) and interstitial lung disease (odds ratio 2.0) were associated with a worse outcome of COVID-19 as individual parameter, although no increased mortality was demonstrated for the IRD patients. Furthermore, 7.1% of patients in the IRD registry smoked compared to 5.5% of patients in the LEOSS registry. The prevalence of smoking in both registries is lower than the smoking prevalence in Germany (2019: 23.8%) (17). A similar result can be shown for the harmful use of alcohol (Germany 2019: 3.1%; IRD registry: 2.7% and LEOSS registry: 0%) (17).

Regarding symptoms of COVID-19, our IRD patients presented significantly more frequently with fever (IRD registry: 70.2% and LEOSS: 38.8%), respiratory symptoms including cough (IRD registry: 62.3% and LEOSS: 39.6%) and dyspnoea (IRD registry: 55.2% and LEOSS: 30.6%). These results are comparable to previously published data (4, 6).

For COVID-19 related death, no significant differences were observed in our cohorts, although IRD patients were significantly more frequently treated with oxygen administration (IRD registry: 72.1% and LEOSS: 51.6%) and non-invasive ventilation (IRD registry: 11.5% and LEOSS: 1.1%) compared to the LEOSS cohort.

This study confirmed known risk factors such as age, male gender in both cohorts. Regarding immunomodulatory therapies rituximab was a significant risk factor for a severe course and poor outcome of COVID-19 in IRD patients, whereas the use of TNF-I was associated with a significantly better outcome in IRD patients, even when compared to the general population without any immune-mediated disease or immunomodulatory treatment. This is an important finding of our analysis, as most of the studies investigating the impact of TNF-I were performed only in IRD patients without employing any comparison to the general population/control group (6, 18, 19). Although the mechanism of SARS-CoV-2 associated hyperinflammation is not fully understood, it is already known that increased TNF concentration in the serum is associated with COVID-19 related organ damage and worse outcome (20). Therefore, blocking of TNF might inhibit COVID-19 related organ damage. A case series already reported beneficial effects of TNF-I in the context of COVID-19 (21) and further studies are ongoing investigating the association of TNF-I and COVID-19 related outcome (22). Reports from the early phase of the pandemic from Wuhan (China) and Boston (USA) reported significant higher risk for severe COVID-19 in IRD patients compared to non-IRD patients, although there was no significant higher rate of death (23, 24). Our data are in accordance with these data, as the COVID-19 related lethality was similar in both registries.

## Strengths and limitations

The strength of our study is the collaboration of two large nationwide registries. Compared to previous studies, data of our study derives from one country with patients treated within the same healthcare system. In addition, until December 2021 (the time period taken for this analysis) no COVID-19 vaccines were available, ruling out the influence of vaccination. To our knowledge, this is the largest study comparing IRD patients to the general population without any immune-mediated diseases, and without any immunomodulatory treatment with detailed clinical information. This allows proper estimation of the role or type of IRD and of immunomodulatory treatments in the context of COVID-19. As the data derived from a phase of the pandemic without any available antiviral treatment, there were no differences in specific COVID-19 treatments.

Limitations of this study include the risk of reporting bias because the registries used convenience sampling. However, our data are in accordance with previous published data, even analysis of health record network, suggesting that reporting bias was not a substantial bias of this study. Although the case report forms were similar, the data were not completely uniform. For example, comorbidities, pre-existing medication were recorded slightly differently across both registries. For this reason, no analysis of former medication was performed. Another limitation concerns the small number of patients who received specific types of immunomodulatory treatment, such as JAK-I. Only few patients with nicotine use were reported which might also be due to reporting bias. For this reason, no conclusion could be drawn regarding the effect of these immunomodulatory treatments and nicotine use in the context of COVID-19. As a further limitation, it should be pointed out, that the study covers the first two waves of the pandemic. SARS-CoV-2 variants and SARS-CoV-2 infection has been changed over time as well as in the clinical presentation of patients. Nevertheless, the study provides an insight into the disease process in patients with IRD and immunosuppressive/immunomodulatory therapy.

## Conclusions

In conclusion, risk factors for increased severity of SARS-CoV-2 infection known for the general population, such as age, male gender, and certain chronic conditions, play a similar role in patients with IRD. Although, chronic kidney disease and interstitial lung disease showed an increased risk for severe COVID-19 compared to the general population, no higher risk for COVID-19 related death could be observed in IRD patients. Regarding treatment, rituximab was associated with worse outcome of COVID-19 and most importantly, our data show that treatment with TNF-I was associated with better outcome of COVID-19 compared to the herewith untreated control group, indicating a protective effect of TNF-inhibition against severe COVID-19 disease during the first two waves of pandemic.

## Data availability statement

The datasets used and/or analysed during the current study are available from the corresponding author on reasonable request.

## Ethics statement

The COVID-19 registry for IRD patients has been approved by the ethics committee of the Justus-Liebig-University Giessen (#52-50), Germany. Approval for the LEOSS registry was obtained by the applicable local ethics committees of all participating centers and registered at the German Clinical Trails Register (DRKS, No. DRKS00021145). For the present analysis, we included data from March 2020 to January 2021. The studies were conducted in accordance with the local legislation and institutional requirements. Written informed consent for participation was not required from the participants or the participants’ legal guardians/next of kin because an analysis of anonymized data from two registries was carried out, where it was not necessary to obtain patient consent due to the ethics votes and the regulations in Germany.

## Author contributions

RH: Writing – original draft, Writing – review & editing. FH: Writing – review & editing, Investigation. MS: Investigation, Data curation, Validation, Writing – original draft. CS: Writing – original draft, Writing – review & editing. TW: Writing – original draft, Writing – review & editing, Data curation, Validation. SB: Writing – review & editing, Investigation. MHa: Investigation, Writing – review & editing. BHe: Investigation, Writing – review & editing. MHo: Investigation, Writing – review & editing. BHo: Investigation, Writing – review & editing. CK: Writing – review & editing, Data curation, Formal Analysis, Methodology, Validation. AK: Investigation, Writing – review & editing. ML-T: Investigation, Writing – review & editing. H-MT: Investigation, Writing – review & editing. UM: Investigation, Writing – review & editing. SM: Investigation, Writing – review & editing. MP: Investigation, Writing – review & editing. AR: Investigation, Writing – review & editing, Data curation, Formal Analysis. JR: Writing – review & editing, Investigation. SR: Investigation, Writing – review & editing. CR: Investigation, Writing – review & editing. MR: Investigation, Writing – review & editing. TS: Investigation, Writing – review & editing. HS-K: Investigation, Writing – review & editing. AS: Investigation, Writing – review & editing. MV: Investigation, Writing – review & editing. FV: Investigation, Writing – review & editing. RV: Investigation, Writing – review & editing. JV: Investigation, Writing – review & editing, Conceptualization, Methodology, Project administration. UM-L: Conceptualization, Investigation, Methodology, Writing – review & editing, Supervision, Writing – original draft. AP: Writing – original draft, Writing – review & editing.

## Footnotes

1
www.covid19-rheuma.de

2
https://leoss.net
